# Non-toxigenic *Corynebacterium diphtheriae* endocarditis: A cluster of five cases

**DOI:** 10.4102/sajid.v39i1.539

**Published:** 2024-02-21

**Authors:** Tamsin Lovelock, Mignon du Plessis, Clinton van der Westhuizen, Jacques T. Janson, Charlene Lawrence, Arifa Parker, Alfonso Pecoraro, Hans Prozesky, Anne von Gottberg, Jantjie Taljaard

**Affiliations:** 1 Division of Infectious Diseases, Department of Medicine, Faculty of Health Sciences, Stellenbosch University and Tygerberg Hospital, Cape Town, South Africa; 2 Centre for Respiratory Diseases and Meningitis, National Institute for Communicable Diseases, Johannesburg, South Africa; 3 School of Pathology, Faculty of Health Sciences, University of the Witwatersrand, Johannesburg, South Africa; 4 Department of Medical Microbiology, Tygerberg Hospital, National Health Laboratory Service, Cape Town, South Africa; 5 Division of Medical Microbiology and Immunology, Department of Pathology, Tygerberg Hospital/Stellenbosch University, Cape Town, South Africa; 6 Division of Cardiothoracic Surgery, Department of Surgery, Tygerberg Hospital/Stellenbosch University, Cape Town, South Africa; 7 Western Cape Government, Department of Health and Wellness, Emergency and Clinical Services Support, Service Priorities Coordination, Communicable Disease Control and Outbreak Response, Cape Town, South Africa; 8 Division of Cardiology, Department of Medicine, Stellenbosch University and Tygerberg Hospital, Cape Town, South Africa

**Keywords:** infective endocarditis, non-toxigenic *Corynebacterium diphtheriae*, outbreak

## Abstract

**Background:**

Classical toxin-mediated respiratory diphtheria has become less common because of widespread effective vaccination globally but invasive disease as a result of non-toxigenic strains of *Corynebacterium diphtheriae* is not prevented by vaccination and may result in severe disease, including infective endocarditis (IE).

**Objectives:**

To describe the outbreak and subsequent investigation of a cluster of five cases of non-toxigenic *C. diphtheriae* endocarditis.

**Method:**

A retrospective observational case series of five cases of non-toxigenic *C. diphtheriae* endocarditis identified in the rural West Coast district of the Western Cape province of South Africa between May 2021 and June 2021.

**Results:**

Non-toxigenic *C. diphtheriae* IE had an aggressive clinical course with high mortality in this cohort. Only one of five patients survived to hospital discharge. The surviving patient received a prompt diagnosis with early surgical intervention but still had a complicated clinical course. Notably, only one case had a pre-existing risk factor for IE, namely a prosthetic valve. Whole genome sequencing of clinical isolates confirmed that all isolates were of the same novel sequence type of non-toxigenic *C. diphtheriae* but despite a thorough investigation no epidemiological link was ever found between the cases.

**Conclusion:**

Non-toxigenic strains of *C. diphtheriae* are less well known but may be highly virulent and cause severe invasive disease.

**Contribution:**

This is the largest cluster of non-toxigenic *C. diphtheriae* IE ever described in South Africa and expands the body of literature on this unusual but possibly emerging infection.

## Introduction

Classical respiratory diphtheria is caused by toxin-producing *Corynebacterium diphtheriae* (or rarely by other toxin-producing *Corynebacterium* species) and has become less prevalent globally because of widespread vaccination in childhood. The diphtheria vaccine is an inactivated toxoid preparation and provides protection from toxin-mediated disease but does not prevent colonisation or infection with non-toxigenic *C. diphtheriae*. Infection with non-toxigenic *C. diphtheriae* most commonly manifests as mild respiratory disease or cutaneous lesions but has emerged as an important cause of more invasive disease in susceptible individuals.^1^

Infective endocarditis (IE) because of *C. diphtheriae* was first described by Howard in the Johns Hopkins Hospital Bulletin in 1893^2^ with a non-toxigenic organism. Invasive infection with non-toxigenic *C. diphtheriae* was described infrequently^3,4^ following this case but has become more prolific in the literature since the 1980s. Infective endocarditis is the most commonly reported presentation, usually appearing as isolated sporadic cases.^5,6,7,8,9,10,11,12,13^ Splenic abscesses,^14^ septic arthritis^15^ and septicemia^6,9,16,17^ because of non-toxigenic *C. diphtheriae* have also been described.

This report describes a cluster of five cases of endocarditis caused by a non-toxigenic strain of *C. diphtheriae*. The cases occurred over a period of 6 weeks between May 2021 and June 2021 in the West Coast district of the Western Cape province of South Africa.

## Methods

The study was a retrospective observational case series describing five cases of non-toxigenic *C. diphtheriae* endocarditis identified in the West Coast district of the Western Cape province of South Africa between May 2021 and June 2021.

Data were collected from the existing clinical and laboratory records for each patient and from documentation generated as part of the outbreak investigation including case investigation forms and line lists compiled by the Western Cape district and provincial public health outbreak response teams.

## Results

### Case presentations

Clinical findings for four individuals with confirmed IE because of non-toxigenic *C. diphtheriae* and a fifth individual with non-toxigenic *C. diphtheriae* bacteraemia and possible IE are summarised in Table 1. Three individuals had documented features of endocarditis on imaging, and one had endocarditis confirmed at autopsy. The remaining individual who presented with fever and cardiac failure was included retrospectively, based on blood culture results. The patient died before cardiac imaging could be performed, and a post-mortem examination was not pursued. In all five cases, the organism was isolated from blood culture and identified using routine laboratory methods at the tertiary care National Health Laboratory Service (NHLS) diagnostic laboratory.

**TABLE 1:** Clinical characteristics of five individuals with non-toxigenic *Corynebacterium diphtheriae* endocarditis.

No.	Age	ender	Date of presentation	Pre-disposing condition/risk factor	Cardiac involvement	Complications	Antibiotic treatment	Surgical management	Outcome
1	13	Male	14 May 2021	Unknown	Unknown	Unknown	Unknown	None	Died
2	25	Male	29 May 2021	None identified	Large vegetation (26 mm) attached to AMVL, moderate MR (findings at echocardiography)	CVA with haemorrhagic transformation	Penicillin G 6 MU IVI 6 hourly Gentamicin 240 mg IVI daily Vancomycin 1.5 g IVI 12 hourly	Posterior fossa decompression Drainage of right frontal intracranial haemorrhage No cardiac surgery	Died
3	24	Male	01 June 2021	Substance abuse (not intravenous)	Endocarditis of mitral and aortic valves with multiple vegetations, largest 12 mm (findings at post-mortem)	CVA Splenic infarct Multi-organ failure	Penicillin G 6 MU IVI 6 hourly Gentamicin 240 mg IVI daily	None	Died
4	38	Female	11 June 2021	Mitral valve replacement	Mitral valve prosthesis-multiple vegetations (findings at echocardiography)	Renal failure Disseminated intravascular coagulation	Ceftriaxone 2 g IVI 12 hourly Penicillin G 6 MU IVI 6 hourly Gentamicin 240 mg IVI daily Vancomycin IVI according to TDM	None	Died
5	14	Female	14 June 2021	None identified	Multiple vegetations on anterior and posterior leaflets of mitral valve (findings on echocardiography and at surgery)	Acute kidney injury	Penicillin G 6 MU IVI 6 hourly Gentamicin 240 mg IVI daily Vancomycin IVI according to TDM	Mitral valve repair and subsequent revision	Survived to discharge, ongoing follow-up

No., number; AMVL, anterior mitral valve leaflet; MR, mitral regurgitation; CVA, cerebrovascular accident; MU, million units; IVI, intravenous infusion; TDM, therapeutic drug monitoring.

The blood culture bottles of these cases were incubated in an automated microbial detection system. Once identified as positive for growth, a Gram stain was performed directly from the blood culture broth. Small Gram-positive bacilli were noticed, and inoculation and subsequent incubation of the broth were carried out on routine growth media (tryptose blood agar, MacConkey agar and Mueller Hinton agar with 5% sheep blood.) Typically, standard biochemical tests are used to identify the cultured isolates to genus level (e.g., *Corynebacterium* species); however because these cases were either known or suspected to have IE, automated biochemical profiling was performed. As invasive *C. diphtheriae* isolates are uncommon, the first two cases were additionally confirmed by matrix assisted laser desorption ionisation-time of flight (MALDI-TOF) spectrometry. Susceptibility testing for penicillin, ceftriaxone and vancomycin was performed on all isolates. Minimum inhibitory concentrations (MICs) were determined using the gradient diffusion method and were interpreted according to the latest breakpoint criteria published by the Clinical and Laboratory Standards Institute (CLSI).^18^ All isolates had intermediate susceptibility to penicillin and full susceptibility to vancomycin, whereas ceftriaxone varied between susceptible and intermediate breakpoints. The MIC for each antibiotic differed by only one double-dilution between the isolates.

All *C. diphtheriae* isolates were submitted to the national reference laboratory at the National Institute for Communicable Diseases (NICD) for confirmation and further characterisation. All isolates were confirmed as non-toxigenic on Elek and polymerase chain reaction (PCR) testing.^19^ Whole-genome sequencing was performed as previously described by Du Plessis et al.^20^ and the five isolates were the same strain, novel sequence type (ST) 885.

Four of the five individuals were residents of a small rural community approximately 180 km north of Cape Town. These four patients presented to the same local district hospital. One patient died shortly after presentation and the other three patients were referred to the next level of care at the general specialist care regional hospital before being transferred to the academic tertiary care institution in Cape Town. The fifth patient resided in a semi-urban setting in the same town as the regional hospital, where she presented before being transferred to the tertiary care facility. We were unable to find an epidemiological link or history of contact with the community or geographical area where the remaining four cases had presented.

*Case 1* was a 13-year-old male who presented to his local district hospital with fever and cardiac failure in mid-May 2021. He had been previously well. His admission diagnosis was documented as acute rheumatic fever. He was transferred to the regional hospital for further care but died shortly after arrival. Laboratory investigations were notable for elevated white blood cell count and C-reactive protein, thrombocytopenia and renal failure. Blood cultures taken on admission yielded non-toxigenic *C. diphtheriae*. This case was identified retrospectively as belonging to the outbreak so more detailed clinical information was not available. No cardiac imaging was performed, and a diagnosis of IE could not be conclusively proven but was considered plausible.

*Case 2* was a 25-year-old male who presented to his local district hospital 15 days after case 1 with fever, fatigue, myalgias and headache. An initial assessment of tick bite fever was made, and the patient was discharged on oral doxycycline. He returned 2 days later with ongoing fever, worsening headache and new-onset confusion and was transferred to the regional hospital for further investigation. Clinical examination revealed a loud pansystolic murmur in keeping with mitral regurgitation. Transthoracic echocardiography confirmed moderate mitral regurgitation and a large vegetation on the anterior leaflet of the mitral valve. Blood cultures were drawn prior to initiating intravenous penicillin and gentamicin and the patient was transferred to the tertiary care cardiology department. Blood cultures yielded *C. diphtheriae*. There were no clinical features of pharyngeal or cutaneous diphtheria.

Following transfer, the patient’s level of consciousness declined, and computed tomography (CT) imaging of the brain confirmed right frontal and cerebellar septic emboli and infarction with haemorrhagic transformation and raised intracranial pressure. An external ventricular drain was inserted. Decompression of the posterior fossa and drainage of the right frontal haemorrhage were performed. The patient was admitted to the neurosurgical intensive care unit postoperatively and significant improvement in neurological status was documented. Intravenous penicillin G 6 million units every 6 h with intravenous gentamicin 240 mg daily was continued. Vancomycin was added because of non-susceptibility to penicillin on the cultured isolate. Vancomycin was dosed according to therapeutic drug monitoring.

Four days after surgery, the patient’s level of consciousness suddenly deteriorated and he became haemodynamically unstable culminating in cardiac arrest. Resuscitation was unsuccessful and he died 16 days after his initial presentation.

*Case 3* was a 24-year-old male who presented to the local district hospital in June 2021 with fever, back pain and jaundice. He gave a history of poly-substance abuse but denied any intravenous injection of drugs. A diagnosis of IE was suspected and empiric treatment with intravenous penicillin G, gentamicin and cloxacillin was commenced. The patient was referred first to the regional hospital and shortly thereafter transferred to tertiary care. Blood cultures yielded *C. diphtheriae*. During his admission, he developed a left hemiplegia and CT imaging confirmed a right middle cerebral artery infarct. He continued to deteriorate, developed multiorgan failure and died 5 days after admission. A postmortem examination confirmed endocarditis of the aortic and mitral valves with multiple obstructing vegetations, the largest of which measured 12 mm. Valve tissue PCR was positive for *C. diphtheriae*. There were no features of pharyngeal or cutaneous diphtheria on antemortem examination or at autopsy.

*Case 4* was a 38-year-old woman who presented to the district referral hospital in mid-June 2021 with a 2-week history of fever, myalgias, lower abdominal pain and diminished effort tolerance. She had undergone mitral valve replacement for rheumatic heart disease 23 years prior. Her postoperative course had been uncomplicated with diligent follow-up for anticoagulation monitoring. On admission to the referral hospital, she was acutely ill and tachycardic with features of biventricular cardiac failure. Biochemistry was remarkable for elevated inflammatory markers, acute kidney injury and prolonged international normalised ratio (on warfarin). Initial transthoracic echocardiography did not show features of prosthetic valve endocarditis. Blood cultures were sent prior to initiating antibiotics (ceftriaxone) and subsequently yielded *C. diphtheriae*. The patient was urgently transferred to the tertiary level hospital for transoesophageal echocardiography and further care.

The patient was admitted to the medical high care unit at the tertiary level hospital. Intravenous penicillin G and vancomycin were initiated; gentamicin was administered for 72 h then withheld because of renal failure. Antibiotics were subsequently changed to penicillin G and ceftriaxone because of worsening renal failure. Transesophageal echocardiography confirmed multiple vegetations on the mitral valve prosthesis. Her clinical course was complicated by worsening renal failure, disseminated intravascular coagulation and pulmonary oedema. She died 11 days after admission and 15 days after her initial presentation to healthcare.

*Case 5* was a 14-year-old female who presented to the local district hospital in June 2021 with a 2-week history of fever, malaise and sore throat. She had been previously well. Although she complained of a sore throat there was no significant pharyngitis or cervical lymphadenopathy. A murmur was detected on clinical examination at the district hospital and blood cultures were sent prior to initiating antibiotics. Blood cultures flagged positive within 72 h with Gram-positive bacilli and the patient was immediately referred to the regional hospital as a suspected *C. diphtheriae* IE. Transthoracic echocardiography confirmed vegetations on the anterior and posterior mitral valve leaflets with moderate to severe mitral regurgitation. The patient was transferred for urgent cardiothoracic surgery and underwent a mitral valve repair procedure within 24 h. She received intravenous penicillin G and vancomycin with therapeutic drug monitoring and completed a total of 42 days of antibiotics. Gentamicin was also initially administered but discontinued after three doses.

The patient developed significant mitral regurgitation postoperatively and required a second mitral valve repair procedure. Her admission was also complicated by acute kidney injury and nosocomial sepsis, but she was discharged home 2 months after initial presentation and continues to follow up with cardiology services.

### Outbreak investigation

**FIGURE 1:**
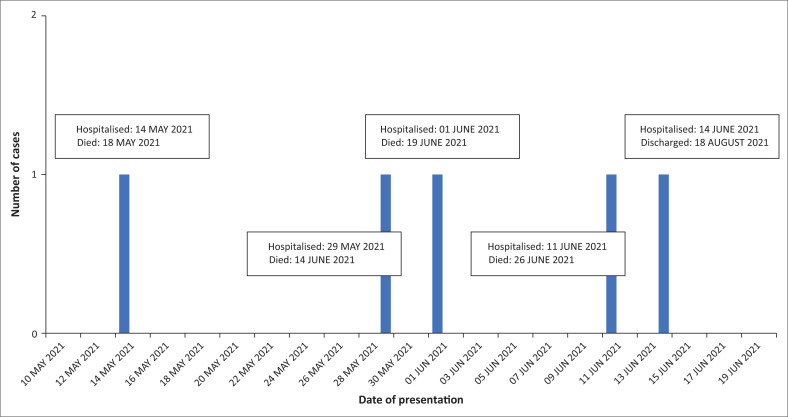
Cases of non-toxigenic *Corynebacterium diphtheria* endocarditis.

Following the identification of the second case of confirmed *C. diphtheriae* endocarditis from the same community, an outbreak investigation was launched by district and provincial public health services. The definitions applied for suspected, probable and confirmed cases and contacts are outlined in Table 2^21^. A timeline of the five cases is presented in Figure 1.

**TABLE 2:** Case definitions applied in the investigation of an outbreak of non-toxigenic *Corynebacterium diphtheriae* endocarditis.

Type of case	Criteria
Suspected case	Individual of any age, resident in the district of interest, presenting with any one of the following: an upper-respiratory tract illness with sore throat and fever an adherent membrane of the nose, pharynx, tonsils, or larynx a chronic skin lesion (scaling rash or ulcer) fever or infection with no clear source symptoms or signs of infective endocarditis symptoms or signs of septic arthritis or osteomyelitis non-specific symptoms with a history of drug use
Probable case	Any case fulfilling criteria for a suspected case with an epidemiological link to a confirmed or suspected case, but no laboratory evidence of *C. diphtheriae*
Confirmed case	Any case with confirmed laboratory evidence of non-toxigenic *C. diphtheriae* (culture or PCR) infection and a compatible clinical picture (as above)
Contact	Any individual fulfilling any one of the following criteria: direct physical contact with a confirmed case residing in the same household as a confirmed case spent significant time with a confirmed case in a workplace or classroom setting

*Source:* Adapted from Western Cape Government. Circular: H91/2021 - Diphtheria alert: Non-toxigenic diphtheria cluster identified in the West Coast and Cape Winelands districts. Annexure 1 [homepage on the Internet]. 2021 [cited n.d.]. Available form: https://www.westerncape.gov.za/assets/departments/health/FP/wcgh_circular_h91_of_2021_-_diphtheria_alert_non-toxigenic_diphtheria_cluster_identified_in_the_west_coast_and_cape_winelands_districts.pdf

PCR, polymerase chain reaction.

Detailed line lists of contacts of each case were generated, and each contact was followed up, tested for *C. diphtheriae* carriage or infection by nasopharyngeal swab and provided with azithromycin prophylaxis after the specimen had been taken. Two cases were school-going children (attending two different schools) and screening of the entire grade of each case was performed. The outbreak occurred during the severe acute respiratory syndrome coronavirus 2 (SARS-CoV-2) pandemic while lockdown measures were in place throughout South Africa and learners were attending school only every second or third day making transmission via the school less likely. A potential additional case may have occurred prior to case 1: a child at the same school, although in a different grade, had died after a sudden illness in the month before case 1 presented. No further medical history surrounding this case could be obtained. No positive contacts were identified through screening of the school contacts.

Detailed line lists were also generated for the adult cases and each contact was tested and provided with antibiotic prophylaxis. A total of 119 nasopharyngeal swabs were submitted for *C. diphtheriae* screening. Swabs were inoculated on tellurite agar and incubated for a total of 72 h. Agar plates were halved to accommodate two swabs per plate. Growth of typical black colonies, suggesting *Corynebacterium* species, was identified by automated biochemical profiling. Gram stains and/or catalase tests were performed prior to biochemical identification if colonies were of doubtful significance (e.g., grey instead of black phenotype). Only one swab was positive for *C. diphtheriae*, confirmed by MALDI-TOF spectrometry. This positive screen was referred to the NICD where testing confirmed the same novel sequence type (ST-885) of *C. diphtheriae* that had been identified in the clinical cases. This specimen belonged to an asymptomatic contact of case 2. This contact was the life partner of case 2 and they had shared a residence.

Careful interviews of contacts and the community failed to identify any clear epidemiological links between the cases regarding residence or places of employment. As a result of lockdown measures, most public recreational areas (restaurants, community centres, places of worship, sports clubs, etc.) were not operating, which limited possible common sources of infection or routes of transmission.

Transmission of *C. diphtheriae* is usually by respiratory droplet and the outbreak investigation considered drugs of abuse to be potentially implicated in the transmission of non-toxigenic *C. diphtheriae* between cases. Only a single case admitted to inhalational drug use but smoking of cannabis was conspicuous within the community affected by the outbreak. A cannabis sample was received by the tertiary care laboratory and was ground in a mortar and pestle before being inoculated in nutrient agar. After 24 h of aerobic incubation, the broth was inoculated on different agars, including tellurite agar. No coryneform bacteria were cultured (screened by Gram stain) and no further specimens were received.

## Discussion

This cluster of cases was notable for the aggressive nature of the infection and high mortality. A recent prospective cohort of 72 IE cases in the Western Cape described an in-hospital mortality rate of 18%^22^ in contrast to the 80% mortality rate observed in our cohort. Only one individual survived, likely because of early identification and prompt surgical intervention (within 24 h of diagnosis). Despite this urgent intervention, this patient still suffered relapse following mitral valve repair, necessitating a second surgical procedure. The high rate of complications and relentless progression of disease despite appropriate, directed antibiotic therapy highlights the importance of surgical management of IE.^22^ Although the pathogenesis of non-toxigenic *C. diphtheriae* infection remains obscure, it seems likely that other virulence factors replace toxin production in these strains. Differences in protein secretion related to synthesis of pili and cellular adherence and invasion may contribute to virulence in some strains.^23^

Another remarkable facet of this outbreak when compared with other outbreaks described in the literature is the absence of a clear cardiac risk factor in four of the five cases. One case involved a patient with prosthetic valve endocarditis, but the remaining patients were assumed to have developed IE on structurally normal cardiac valves. A cluster of seven cases of endocarditis because of non-toxigenic *C. diphtheriae* that occurred in New South Wales, Australia, over a 12-month period was described in 1992 by Tiley et al.^10^ The cohort was notable for the aggressive course of disease with significant morbidity as well as the presence of septic arthritis in association with endocarditis in four of the seven cases. Three cases had no identifiable underlying risk factor for endocarditis.

A single case in our cohort gave a history of substance abuse and poor living circumstances. This has been associated with invasive disease with non-toxigenic *C. diphtheriae* in other cohorts,^17^ but not with *C. diphtheriae* IE. A series of seven cases (occurring over a 5-year period) of non-toxigenic *C. diphtheriae* bacteraemia was identified in Vancouver, Canada and described in 2005 by Romney et al.^17^ Lower socioeconomic status, poor living conditions and substance dependence, particularly alcoholism, were identified as risk factors. The presence of skin lesions in several cases was a likely portal of entry for invasive infection. No endocarditis was described in this series.

Intravenous or inhaled drugs were considered as a potential point source of the outbreak but only one patient reported any use of illicit substances. This patient strenuously denied any intravenous drug use. An association between *C. diphtheriae* bacteraemia and intravenous drug use was demonstrated in a review by Gubler et al.^9^ Fourteen cases of non-toxigenic *C. diphtheriae* bacteraemia were identified in Switzerland between 1990 and 1996. Of these, 12 cases reported intravenous drug use and frequented the same area in Zurich to acquire and inject intravenous drugs. Nine cases had proven endocarditis, which was associated with high mortality. Ribotyping of isolates revealed that 13 of the 14 cases had been caused by the same clone of non-toxigenic *C. diphtheriae* var. *mitis*. Cases of invasive *C. diphtheriae* infection declined after the open drug scene in Zurich was dissolved in 1995.

All cases in our cohort were infected with the same novel strain (ST-885) of non-toxigenic *C. diphtheriae*. Compared with other *C. diphtheriae* organisms collected previously in South Africa, this strain was most closely related to non-toxigenic ST-390 (triple-locus variant), also isolated from an endocarditis patient in 2015 in the same province.^20^ Sequence type 885 is also a triple-locus variant of ST-395: a non-toxigenic strain that was detected during the 2015 diphtheria outbreak investigation in KwaZulu-Natal province.^20^ A common source of infection or chain of transmission seems most likely, but no clear epidemiological link between cases could ever be identified and one case occurred in a geographical area at a distance of almost 150 km from the other four cases. The only shared contact point prior to admission to the tertiary level hospital was the specialist referral hospital for the district, but no two cases were ever admitted simultaneously, and all cases had established endocarditis and *C. diphtheriae* infection proven on blood cultures, which were taken on admission or soon thereafter, excluding nosocomial infection.

*Corynebacterium diphtheriae* is transmitted primarily by respiratory droplet spread and one individual with asymptomatic nasopharyngeal carriage of ST-885 was identified with contact tracing. Other unidentified asymptomatic carriers may have been the means of transmission through the community. The abrupt onset and short duration of the outbreak remain unexplained, but it is possible that the outbreak may have been halted by masking and social distancing implemented as part of SARS-CoV-2 lockdown measures. Previous cases may have been missed because of *Corynebacterium* species being dismissed as skin commensals and contaminants in blood culture processing but for several months following the outbreak all *Corynebacterium* species on blood culture were followed up and no further cases came to light.

## Conclusion

These unusual cases of highly invasive disease because of non-toxigenic *C. diphtheriae* represent a departure from the expected spectrum of vaccine-preventable, toxin-mediated disease usually associated with *C. diphtheriae. Corynebacterium* species may not always be regarded as clinically significant when isolated from clinical specimens outside of the respiratory tract, and further speciation and identification are not always pursued, especially if the clinical picture is not that of typical respiratory diphtheria. As a result, these infections may never be identified and their burden of disease may be underestimated. Portals of entry, mechanisms of spread and host susceptibility factors for invasive *C. diphtheriae* disease remain poorly characterised and may be of particular importance in the setting of an outbreak.

When *C. diphtheriae* is identified, there is a strong clinical imperative to determine if the strain is toxin-producing because of the public health implications and potential benefit of treating with antitoxin. The aggressive nature of invasive infection with non-toxigenic strains demonstrated in this cohort suggests that the organism bears alternative virulence factors to toxin production and reminds us that non-toxigenic *C. diphtheriae* infections should not be underestimated.
